# Perceiving a Stranger's Voice as Being One's Own: A ‘Rubber Voice’ Illusion?

**DOI:** 10.1371/journal.pone.0018655

**Published:** 2011-04-07

**Authors:** Zane Z. Zheng, Ewen N. MacDonald, Kevin G. Munhall, Ingrid S. Johnsrude

**Affiliations:** 1 Centre for Neuroscience Studies, Queen's University, Kingston, Canada; 2 Department of Psychology, Queen's University, Kingston, Canada; 3 Department of Otolaryngology, Queen's University, Kingston, Canada; French National Centre for Scientific Research, France

## Abstract

We describe an illusion in which a stranger's voice, when presented as the auditory concomitant of a participant's own speech, is perceived as a modified version of their own voice. When the congruence between utterance and feedback breaks down, the illusion is also broken. Compared to a baseline condition in which participants heard their own voice as feedback, hearing a stranger's voice induced robust changes in the fundamental frequency (F0) of their production. Moreover, the shift in F0 appears to be feedback dependent, since shift patterns depended reliably on the relationship between the participant's own F0 and the stranger-voice F0. The shift in F0 was evident both when the illusion was present and after it was broken, suggesting that auditory feedback from production may be used separately for self-recognition and for vocal motor control. Our findings indicate that self-recognition of voices, like other body attributes, is malleable and context dependent.

## Introduction

The rubber hand illusion describes a phenomenon where temporally coincident visual and somatosensory inputs (i.e., the feeling of someone stroking the fingers of your own hidden hand, and the sight of a prosthetic hand being stroked the same way) combine to create the perception of body ownership, a critical component of self-awareness [1], [2]. Similar perceptual illusions have also been demonstrated with respect to the face [3], and the whole body [4]. It has been argued that self-attribution of body parts is mediated by multisensory perceptual correlations [1], [5], [6]. This means that the illusion itself is not modality specific and similar phenomena might be observed across other modalities, as long as multi-modal cues are converging.

Behavioral studies have documented illusory self-attribution during voluntary action when action-related sensory cues were manipulated to be coherent and congruent [7]–[9]. For example, Van den Bos & Jeannerod [9] demonstrated that when an experimenter's hand was the only visual information presented to the participants and when that experimenter performed, in synchrony, the same finger movements as the participants did, participants tended to identify the experimenter's hand as their own hand. This series of studies can be taken as evidence that motor information provided during action can modulate the perception of body ownership when the motor movement and its sensory consequences are consistent [10].

Although there is a growing literature on the perception of body ownership, empirical evidence regarding the perception of voice ownership as a result of vocal motor output is lacking. Vocal production provides rich sensory feedback signals (i.e., auditory and somatosensory) which, together with representations generated during articulation, can contribute to the recognition of one's own voice through a self-monitoring system [11], [12]. Previous studies have shown that psychotic patients with positive symptoms of auditory hallucinations and delusions of control have difficulty identifying self-produced sounds, and this appears to be due to an impaired self-monitoring system [13]–[15]. However, it remains unclear how normal individuals, in whom the self-monitoring system is intact, would perceive the identity of an external voice that is heard as concomitant auditory feedback of their own vocalizations.

An important aspect of auditory feedback during vocal production is that it is used for vocal motor control of ongoing speech (e.g., [16]). Based on online feedback perturbation paradigms, a number of studies have either provided behavioral [17]–[19] or neuroimaging [20]–[23] evidence for the role of auditory feedback in articulatory control, as part of an error correction mechanism. The question that remains to be addressed here, however, is whether auditory feedback is used the same way for vocal motor control as for the recognition of one's own voice.

To address these issues, we examined, both subjectively and objectively, how normal participants responded to a feedback voice that was heard as the auditory concomitant of their own vocalizations. Specifically, participants produced one of two target words on each trial, and heard auditory feedback temporally gated with their own utterances using a real-time signal processing system. We assessed participants' subjective perception of, and vocal-motor adaptation to, online auditory feedback of a) their own voice, and b) one of two stranger voices, during vocal production. We were interested in exploring how participants would perceive the stranger voice feedback both when it was congruent with their own production and when it was not, as well as how participants' subjective perception of voice identity was related to the acoustics of their vocal production.

## Method

### Participants

Ninety-three right-handed female participants (age range: 18–28 years, mean: 22 years) recruited from the Queen's community participated in this study. Participants were without any history of neurological or hearing impairment, and spoke English as their first language. Written informed consent was obtained from all human participants. All procedures were cleared by the Queen's General Research Ethics Board.

### Materials

Two female speakers of southern Ontario English were recruited as stimulus voices (V1 and V2). Prior to the experiment, utterances of the target words, ‘day’ and ‘too’, from these two stimulus voices were recorded in a soundproof booth. The two target words were selected because they are one-syllable English words of consonant-vowel (CV) form. A pilot study demonstrated that temporal aspects of production are similar across talkers. Utterances of two additional words, ‘page’ and ‘test’, were also recorded. The individuals recruited as stimulus voices were chosen because the pitch of their voices was either higher (V1) or lower (V2) than that of an average female talker of southern Ontario English. MacDonald et al. [24] reported that for the vowels/e/and/u/, the F0 of an average female talker was 204 and 213 Hz, respectively. For the utterances used in this study, V1 had an F0 of 226 and 241 Hz for/e/and/u/respectively; V2 had an F0 of 187 and 200 Hz for/e/and/u/.

### Procedure

Testing took place in a soundproof booth with a microphone (Sennheiser E845S, Sennheiser Electronic, Germany) and a set of headphones (Sennheiser HD265 Linear, Sennheiser Electronic, Germany) connected through a Fireface 400 audio interface (RME, Germany) to a real-time signal processing computer (National Instruments, TX), on which a deterministic signal processing program was implemented [25], [26]. This real-time system is capable of delivering auditory stimuli, either pre-recorded or relayed directly from the microphone, through the headphones without noticeable delay (iteration delay less than 10 ms) from the onset of speech production. In the cases when participants' utterances were shorter than the recorded stimulus voice utterances, our system would match the offset of their vocalizations by truncating the recorded utterances after production ceased.

The experiment consisted of 155 trials. On each trial, participants were prompted to speak either ‘day’ or ‘too’ into the microphone and heard concomitant auditory feedback through the headphones. Low-level white noise was present in the headphones to minimize the bone-conducted speech feedback while they vocalized [27]. Performance on each trial was monitored by the experimenter (ZZZ) from outside the booth and also recorded by our system. Over the 155 trials, 80 trials of ‘day’ and 75 trials of ‘too’ were presented in the same pseudorandom order for each participant, such that no more than three consecutive trials of the same word occurred.

#### a) Experimental paradigm and groups (Early Mismatch and Late Mismatch)

**Figure 1:**
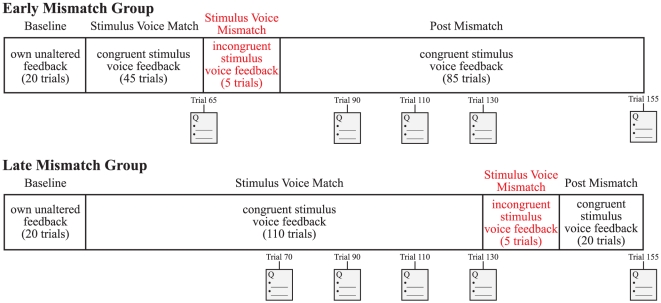
Schematic diagram of the four stages of the experiment. See text for details.

The experiment consisted of 4 stages: Baseline, Stimulus Voice Match, Stimulus Voice Mismatch, and Post Mismatch (see Figure 1). During the Baseline stage, participants produced 20 utterances of the target words while receiving their own unaltered feedback. During the three other stages, when participants produced a target word, they heard one of the utterances produced by a stimulus voice (V1 or V2). This feedback matched the produced word in the Match and Post Mismatch stages, but differed in the Mismatch stage where participants produced ‘day’ or ‘too’ but heard the stimulus voice saying ‘page’ or ‘test’.

Participants were randomly assigned into one of two experimental groups (Early Mismatch and Late Mismatch) that differed in when the Mismatch stage occurred. The Match stage consisted of 45 trials (Early Mismatch) or 110 trials (Late Mismatch). The Mismatch stage occurred at trials 66–70 (Early Mismatch) or trials 131–135 (Late Mismatch). The final stage, Post Mismatch, was similar to the Match stage, but consisted of 20 and 85 trials for the Late- and Early Mismatch groups respectively.

#### b) Subjective report

At five time points over the course of the experiment (see Figure 1), participants responded to two questions concerning the perceived identity of the feedback voice using a 7-point Likert scale in the form of a sliding pointer on the computer screen (with 1 indicating “strongly disagree” and 7 indicating “strongly agree”). The two questions were adapted from the rubber voice illusion questionnaire [1]: 1) “It felt as if the voice I heard was my voice”, and 2) “It felt as if the voice I heard was a modified version of my voice”. For the Early Mismatch group, these questions were asked once before and four times after the Mismatch stage, whereas for the Late Mismatch group, they were asked four times before and once after the Mismatch stage. Responses were measured to the nearest 0.1 units.

**Figure 2:**
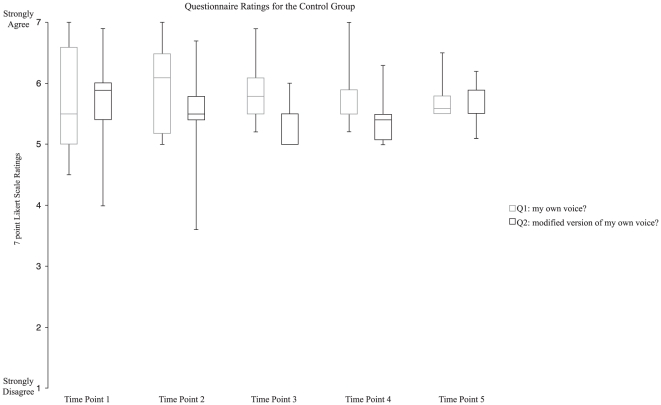
The box plots for ratings on the two questions (Q1 and Q2) across five time points are shown for a small control group (N = 5).

To test the validity of our questions, five participants had their own voice utterances recorded during the Baseline stage. These own utterances were then used as stimulus voice feedback during the remaining 135 trials of the experiment (i.e., feedback always temporally gated and congruent with vocalizations). As expected, these participants rated both Q1 and Q2 high across the five time points (see Figure 2). This suggests that these two question items are not mutually exclusive.

However, a trivial reason for people reporting that the stimulus voice was like their own voice would be that they could not distinguish between the two. We examined this in a separate group of 10 participants tested in a pilot version of the procedure reported here. After the procedure, their ability to distinguish their own recorded productions of five monosyllabic words (including ‘day’ and ‘too’) from those of the stimulus voice was tested on 20 trials. In each trial, one monosyllabic word was presented twice; once in their voice and once in the stimulus voice. They were asked to report the interval in which their own voice was presented. Participants in this study were all able to distinguish their own voices from the stimulus voice with 100% accuracy. This result indicates that any perceptual effects in our study cannot be due simply to the inability of our participants to distinguish between their own and the stimulus voice.

#### c) Vocal production data analysis

We extracted the fundamental frequency (F0) across the 155 trials for each participant in order to track the acoustics of produced vowels (see [25], [26]). The F0 track for every trial from each participant was individually reviewed for discontinuities and/or gaps caused by glottal fry using Praat [28]. Trials with glottal fry were excluded from analysis, as were participants who 1) exhibited glottal fry on more than 30% of baseline trials (>6 trials) or more than 20% of the entire trials (>30 trials); 2) produced sounds (e.g., coughs) that resulted in unplanned mismatches in feedback; or 3) had an F0 that was higher than the stimulus voice for one target word but lower for the other. As a result of these exclusion criteria, 29 and 33 participants were included in the Early- and Late Mismatch groups respectively.

The F0 data for each participant were then examined to determine whether vocal production shifted up, or down, or not at all during stimulus voice feedback, relative to baseline. For each word, the mean and standard error of the last five trials of the Baseline (excluding the first 5 trials of each word to allow the participant to adapt to the microphone) were used to determine the 95% confidence interval (CI). If the mean F0 of the 10 trials immediately preceding the Mismatch stage fell within the baseline 95% CI, the participant was classified as ‘no shift’; if the F0 was outside the 95% CI, the participant was classified as ‘up’ if it was higher and ‘down’ if it was lower. In addition, for every trial after the Baseline stage, for each participant, a normalized F0 shift (relative to baseline) was calculated by subtracting the mean baseline F0 for each word separately.

## Results

### a) Subjective ratings

**Figure 3:**
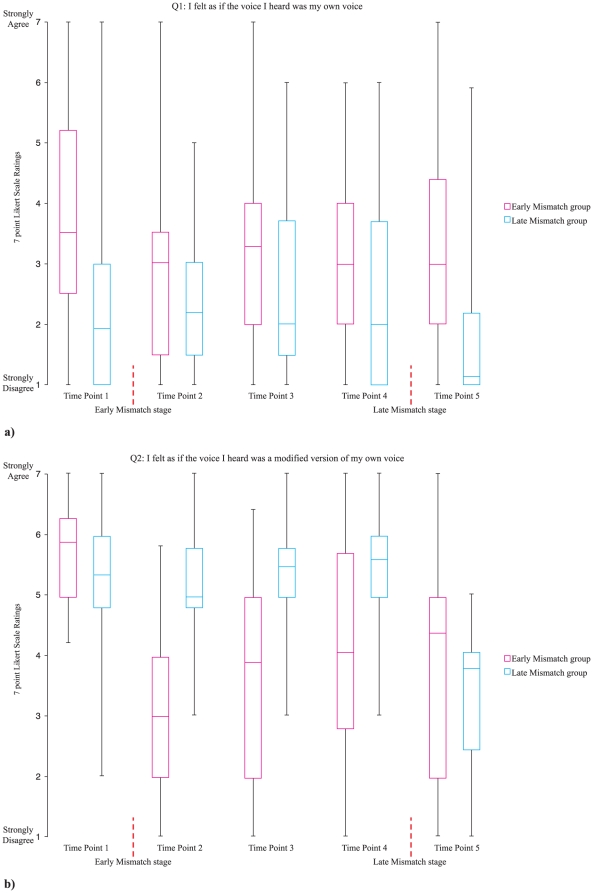
The box plots for ratings on the two questions (Q1 and Q2) are shown for the Early Mismatch and Late Mismatch group. a) Question 1: I felt as if the voice I heard was my own voice, and b) Question 2: I felt as if the voice I heard was a modified version of my own voice, were rated on a 7-point Likert scale across five time points. The Mismatch stage occurs after the first time point for the Early Mismatch group and after the fourth time point for the Late Mismatch group, as indicated by a red vertical dashed line.

In general, ratings were low across all time points for Q1 (i.e., “I felt as if the voice I heard was my own voice”), but high before and low after the Mismatch stage for Q2 (i.e., “I felt as if the voice I heard was a modified version of my own voice”) (see Figure 3).

These observations were confirmed with MANOVAs on the ratings on each of the two questions across the five time points, with stimulus voice (V1 and V2) and group (Early Mismatch and Late Mismatch) as between-subjects factors. For Q1, a pattern of results consistent with the mismatch events having a marked effect on ratings was rather weakly observed. There was a marginally significant interaction between time and group *F*(4, 55)  = 2.52, *p* = .052, η_p_^2^ = .16, with ratings being higher in the Early Mismatch group than in the Late Mismatch group at both first and last time points, *p* = .001 and *p* = .002, respectively. However, ratings dropped significantly after the Mismatch only in the Early Mismatch group, *p* = .004.

In addition, we observed a higher overall rating in the Early Mismatch compared to the Late Mismatch group, *F*(1, 58)  = 8.56, *p* = .005, η_p_^2^ = .13, and a marginally significant effect of time, *F*(4, 55)  = 2.47, *p* = .055, η_p_^2^ = .15. A trend analysis indicated that there was a cubic trend of ratings across the time points, *F*(1, 58)  = 8.04, *p* = .006, η_p_^2^ = .12, such that ratings decreased after the first time point, and then started to gradually increase, before decreasing again at the last time point.

Sign tests on Q1 ratings at each of the time points indicated that for the Early Mismatch group, the ratings at the first and last time points were not different from ‘neutral’ (i.e., a rating of ‘4’), *p* ≥.061, but below ‘neutral’ at time points 2, 3, and 4, *p*≤.008. For the Last Mismatch group, the ratings were below ‘neutral’ at all time points, *p*≤.001.

The three-factor MANOVA on Q2 ratings revealed a strong interaction between time and group *F*(4, 55)  = 25.26, *p*<.001, η_p_^2^ = .65. Follow-up pairwise comparisons with Bonferroni correction revealed that, for the Early Mismatch group, Q2 ratings at time point 1 (pre-Mismatch) were significantly higher than those at all the later time points (post-Mismatch), *p*<.001. In this group, Q2 rating at time point 2 was also lower than that at time point 4, *p* = .003. For the Late Mismatch group, Q2 ratings at the first four time points (pre-Mismatch) were all significantly higher than that at the last time point (post-Mismatch), *p*<.001. The ratings of the two groups did not differ at either the first or last time point (i.e., before the mismatch event for both groups or after it, *p*≥.100) but the ratings from the Late Mismatch group were significantly higher than those of the Early Mismatch group at time points 2, 3, and 4, *p*≤.001, which are pre-Mismatch stage for the Late Mismatch group but post-Mismatch for the Early group (see Figure 3b).

In addition to this expected interaction, participants who heard V1 gave higher ratings that those who heard V2, *F*(1, 58)  = 5.63, *p* = .021, η_p_^2^ = .09, and participants in the Late Mismatch group gave higher ratings than the Early Mismatch group, *F*(1, 58)  = 9.81, *p* = .003, η_p_^2^ = .15. Finally, ratings varied across the time points, *F*(4, 55)  = 34.39, *p*<.001, η_p_^2^ = .71. A trend analysis revealed a combination of linear, *F*(1, 58)  = 33.43, *p*<.001, η_p_^2^ = .37, and cubic, *F*(1, 58)  = 88.65, *p*<.001, η_p_^2^ = .60, components for ratings across the time points, such that ratings dropped after the first time point, and then slowly increased from time point 2 to 4, before dropping again at the last time point.

Sign tests on Q2 ratings indicated that, for the Early Mismatch group, Q2 ratings at the first time point (pre-Mismatch) were reliably greater than ‘neutral’, *p*<.001, but ratings dropped to well below ‘neutral’ at time point 2, *p* = .001 (post-Mismatch) and then were not different from ‘neutral’ for time points 3, 4, and 5, *p*≥.458. For the Late Mismatch group, Q2 ratings at the four pre-Mismatch time points were all reliably greater than ‘neutral’, *p*≤.001, whereas the rating at the post-Mismatch time point dropped to well below ‘neutral’, *p* = .014.

The results, particularly from Q2, suggest that the Mismatch stage, characterized by incongruent stimulus voice feedback, appeared to disrupt the illusion of the stimulus voice being attributed to the ‘self’, as evidenced by altered ratings. Higher Q2 ratings at the later time points for the Early Mismatch group (see Figure 3b) may indicate that after many further trials of congruent feedback, the illusory percept appeared to build again. Overall, it seems that the perceptual illusion regarding the perceived identity of the feedback voice is elicited by congruent feedback, matched in timing and content to the participant’s own vocalization.

#### b) Vocal motor adaptation

**Figure 4:**
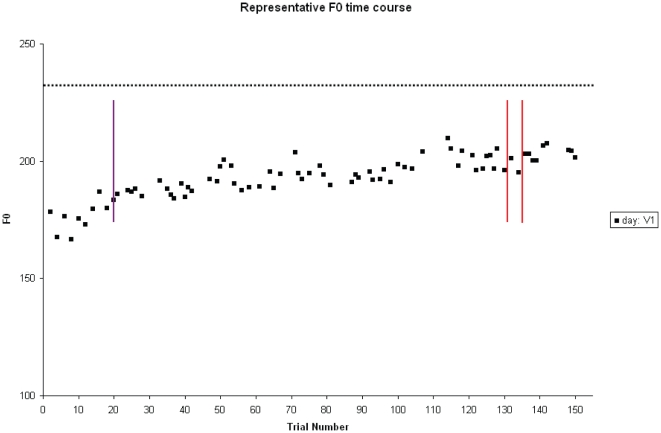
The F0 (Hz) time course for ‘day’ from one representative participant is shown. This participant was from the Last Mismatch group and assigned V1 as the stimulus voice. The solid purple vertical line at trial 20 indicates the end of the Baseline stage. The two solid red vertical lines indicate the beginning and end of the Stimulus Voice Mismatch stage. The black dashed horizontal line indicates the F0 of the stimulus voice V1.

To determine whether and how participants altered production in response to the stimulus voice feedback, we examined the F0 of participants’ vocal production over the course of the experiment (see Table 1). Chi-square tests revealed that significantly more participants altered their F0 than did not change when hearing either V1, χ^2^(1, N = 32) ≥ 10.13, *p*≤.001, or V2, χ^2^(1, N = 30)  = 6.53, *p* = .011. The direction of change depended on the relation between the participant’s baseline F0 and the stimulus voice F0, such that participants were more likely to shift their F0 towards (i.e., ‘follow’), than away from (i.e., ‘compensate’), that of the stimulus voice for both V1, χ^2^(1, N = 32) ≥ 6.76, *p*≤.009, and V2, χ^2^(1, N = 30) ≥ 4.55, *p*≤.033 (see Table 1). Thus participants tended to shift their F0 upward if their F0 was lower than that of the stimulus voice, and downward if their F0 was higher (e.g., see Figure 4).

**Table 1:** The number of participants who shifted their F0 up (Up), down (Down), or did not shift their F0 (No-shift) when hearing either V1 or V2 are shown for ‘day’ and ‘too’.

**day**	Up	Down	No-shift	Follow	Compensate	No-shift	Total
V1	22	4	6	22	4	6	32
V2	16	6	8	17	5	8	30
**too**	Up	Down	No-shift	Follow	Compensate	No-shift	Total
V1	19	6	7	19	6	7	32
V2	13	9	8	16	6	8	30

Each participant was assessed based on whether the average F0 of 10 trials immediately preceding the Mismatch stage was higher than (Up), lower than (Down), or inside (No-shift) the range defined by the 95% CI for the mean baseline F0 (see Procedure c for a more detailed description). For those participants who significantly shifted their F0, the direction of the shift was also determined as to whether the shift was towards (Follow) or away from (Compensate) the stimulus voice heard.

To understand the effect of the Mismatch stage on the pattern of changes in F0, we compared the magnitude of normalized F0 shifts *before* and *after* the Stimulus Voice Mismatch stage for the two groups. Here we used, for each word, the mean magnitudes of F0 shifts during the last 10 trials in the pre-Mismatch and the first 10 trials in the post-Mismatch stages as estimates of *before* and *after* F0 shifts respectively. We conducted a MANOVA with stage (*before* and *after)* and word (‘day’ and ‘too’) as repeated measures, and stimulus voice (V1 and V2) and group (Early Mismatch and Late Mismatch) as between-subjects factors. We observed a higher magnitude of F0 shifts in the *after* stage than in the *before* stage, *F*(1, 58)  = 8.00, *p* = .006, η_p_^2^ = .12. We also observed a three-way interaction between stage, stimulus voice, and group, *F*(1, 58)  = 6.34, *p* = .015, η_p_^2^ = .10. Post-hoc comparisons with Bonferroni correction indicated that the magnitude of F0 shifts was the same *before* and *after* the Mismatch stage for V1 in both groups, *p*≥.288, but greater *after* than *before* the Mismatch stage for V2, *p*≤.042. The observation that F0 shift was either unaltered or even greater after the Mismatch stage, is in sharp contrast to the pattern of subjective ratings. If production data mirrored the subjective ratings, F0 shifts should have been diminished when the illusion was broken.

In addition, the magnitude of F0 shift was higher in the Late Mismatch group than in the Early Mismatch group, *F*(1, 58)  = 4.43, *p* = .040, η_p_^2^ = .07. There was also an interaction between group and voice, *F*(1, 58)  = 4.32, *p* = .042, η_p_^2^ = .07, such that the magnitude was higher in the Late Mismatch group only for V1, *p* = .004.

## Discussion

We believe that the emergence of this auditory illusion results from the convergence of sensory cues in the context of voluntary action. Previous studies have shown that motor action significantly contributes to the self-recognition process by structuring the perception of bodily multisensory signals [9], [10], [29]. The coherence between motor movement and its sensory consequences plays a critical role in modulating the perceptual experience of both body ownership and movement agency [7], [30]. In the present study, auditory feedback was temporally and phonetically congruent with motor and somatosensory feedback from the articulators. The alignment between vocal motor movement and the resulting sensory events allowed the participants to perceptually categorize a stranger's voice as being from themselves, suggesting that the coherent action-related cues are integrated into a unified sense of voice ownership.

It has been suggested that the induction of ownership in the rubber hand illusion depends critically on a top-down process of evaluating the rubber hand against a pre-existing cognitive representation of the body, based on whether the rubber hand is a plausible substitute for the body part [31]. This explains why using a piece of wood [31], a wooden hand [32], or even a rubber hand covered with non-natural skin texture [33] either reduces or abolishes the illusion, depending on the degree of implausibility. Similarly, the stranger's voice in our study, although gender-matched to the participant's own voice, was otherwise very different, and may therefore have been perceived as a somewhat implausible substitute. This may be why participants endorsed Q2 rather than Q1. The large variances in the Q1 ratings may reflect variability in the degree of judged implausibility across individuals.

In addition to the subjective ratings indicating that participants were experiencing illusory ownership of the feedback voice, a trial-by-trial assessment of vocal production revealed that participants shifted their F0 to follow that of the feedback voice. A line of studies using online F0 shift paradigms have shown that, in general, people compensate for a change of F0 in their auditory feedback during vocal production, i.e., by shifting their production in the direction opposite to the shifted feedback signal [17], [19], [34]. Although the central mechanism underlying the direction of vocal motor adaptation is not well understood, the shift must reflect the operation of a sensorimotor control process involved in regulation of ongoing speech production. That our participants tended to follow, rather than compensate, might be due to a number of factors. One factor might be related to the large magnitude of effective ‘shift’ between participants' own voice F0 and the stimulus voice F0, which is within the range over which Burnett et al. [17] observed the greatest proportion of following responses as a result of F0 perturbation.

The observation that F0 remains shifted despite a change in perception of the feedback voice identity suggests a divergence between conscious perception and sensorimotor control. This is consistent with a two-level model of self-action recognition, which posits that an automatic level of action control and a conscious level involving the perception of action agency can be separated [35]. Empirical studies involving online sensory perturbation during motor movements have revealed both mismatch [36] and temporal lags [37] between objective motor responses and subjective awareness of the perturbation. Data supporting such a divergence also comes from clinical studies of patients with visual form agnosia who demonstrate striking precision of hand movements towards a visual target that they fail to perceive [38], and of patients with schizophrenia who are capable of initiating an action but are impaired in attributing the action to its correct source [39]. Our data further add to this literature in demonstrating that the cognitive systems that process auditory feedback for the differentiation between ‘self’ and ‘other’ and for control of ongoing vocal production appear to be at least partially dissociable.

In summary, our study provides a new framework to explore the sense of ownership of voice by examining both perceptual judgment of voice identity, and acoustics of vocal production, in the same context. Our findings shed new light on how identity and acoustic information of voice are processed during talking, and are relevant to the understanding of clinical conditions involving impaired voice ownership attribution such as schizophrenia.
